# Efficacy of high-flow nasal oxygenation against peri- and post-procedural hypoxemia in patients with obesity: a meta-analysis of randomized controlled trials

**DOI:** 10.1038/s41598-022-10396-5

**Published:** 2022-04-19

**Authors:** Kuo-Chuan Hung, Ching-Chung Ko, Po-Chih Chang, Kuei-Fen Wang, I.-Chia Teng, Chien-Hung Lin, Ping-Wen Huang, Cheuk-Kwan Sun

**Affiliations:** 1 Department of Anesthesiology, Chi Mei Medical Center, Tainan City, Taiwan; 2 Department of Hospital and Health Care Administration, College of Recreation and Health Management, Chia Nan University of Pharmacy and Science, Tainan City, Taiwan; 3 Department of Medical Imaging, Chi Mei Medical Center, Tainan City, Taiwan; 4 Department of Health and Nutrition, Chia Nan University of Pharmacy and Science, Tainan City, Taiwan; 5 Division of Thoracic Surgery, Department of Surgery, Kaohsiung Medical University Hospital/Kaohsiung Medical University, Kaohsiung City, Taiwan; 6 Weight Management Center, Kaohsiung Medical University Hospital/Kaohsiung Medical University, Kaohsiung City, Taiwan; 7 Department of Sports Medicine, College of Medicine, Kaohsiung Medical University, Kaohsiung City, Taiwan; 8 Ph. D. Program in Biomedical Engineering, College of Medicine, Kaohsiung Medical University, Kaohsiung City, Taiwan; 9 Department of Emergency Medicine, Show Chwan Memorial Hospital, Changhua City, Taiwan; 10 Department of Emergency Medicine, E-Da Hospital, No.1, Yida Road, Jiaosu Village, Yanchao District, Kaohsiung City, 82445 Taiwan; 11 College of Medicine, I-Shou University, Kaohsiung City, Taiwan

**Keywords:** Health care, Medical research

## Abstract

This meta-analysis aimed at investigating the efficacy of high-flow nasal oxygenation (HFNO) against hypoxemia in patients with obesity compared with conventional oxygenation therapy and non-invasive ventilation. Databases were searched from inception to August 2021. Studies involving peri- or post-procedural use of HFNO were included. The primary outcome was risk of hypoxemia, while the secondary outcomes included status of oxygenation and carbon dioxide elimination. Ten randomized controlled trials (RCTs) were included. We found that HFNO prolonged the safe apnea time at induction compared to control group [mean difference (MD) = 73.88 s, *p* = 0.0004; 2 RCTs] with no difference in risk of peri-procedural hypoxemia [relative risk (RR) = 0.91, *p* = 0.64; 4 RCTs], minimum SpO2 (MD = 0.09%, *p* = 0.95; 4 RCTs), PaO2 (MD = − 8.13 mmHg, *p* = 0.86; 3 RCTs), PaCO2 (MD = − 6.71%, *p* = 0.2; 2 RCTs), EtCO2 (MD = − 0.28 mmHg, *p* = 0.8; 4 RCTs) between the two groups. HFNO also did not improve postprocedural PaO2/FiO2 ratio (MD = 41.76, *p* = 0.58; 2 RCTs) and PaCO2 (MD = − 2.68 mmHg, *p* = 0.07; 2 RCTs). This meta-analysis demonstrated that the use of HFNO may be associated with a longer safe apnea time without beneficial impact on the risk of hypoxemia, oxygenation, and CO2 elimination in patients with obesity. The limited number of trials warranted further large-scale studies to support our findings.

## Introduction

Peri- or post-procedural oxygen supplementation has been a widely accepted approach to increasing pulmonary oxygen reserves and delaying the onset of oxygen desaturation during apnea^1–3^. Oxygen supplementation is essential for patients at induction of anesthesia or receiving sedation, especially those at risk of difficult intubation^4,5^ and those undergoing rapid sequence induction^6^ or with limited oxygen reserves^7^. Oxygen supplementation is also important following anesthesia or administration of sedatives, as the residual effects of these regimens can lead to hypoxemia, hypoventilation, and loss of airway patency^3,8^. Patients with obesity are considered at higher risks of difficulty in mask ventilation^9^ and tracheal intubation^10^ compared with those in individuals without. Besides, obesity is associated with a reduced functional residual capacity (FRC), atelectasis, and significant shunting in dependent lung regions with an increase in resting metabolic rate, work of breathing, and minute oxygen demand^11^. Therefore, patients with obesity are at high risk of a rapid drop in arterial oxygen level after the cessation of breathing. Moreover, because coexisting cardiovascular diseases are common in patients with obesity^12^, hypoxemia-induced cardiovascular complications (e.g., myocardial depression) following sedation remain a major concern^13^. Consequently, effective oxygen supplementation is crucial to the prevention of peri- and post-procedural pulmonary and cardiovascular complications in this patient population.

High-flow nasal oxygenation (HFNO) refers to the delivery of oxygen at high flow rates without recourse to invasive or non-invasive ventilation. In the critical care setting, pooled evidence has demonstrated the clinical benefits of applying HFNO in patients with acute respiratory failure or those at high risk of post-extubation respiratory failure^14–16^. A recent meta-analysis recruiting mostly patients without obesity also reported the effectiveness of HFNO for prolonging the duration of safe apnea and elevating minimum SpO_2_ as well as decreasing the risk of hypoxemia in patients receiving anesthetic induction or sedation^17^. Because upper airway obstruction due to posterior displacements of oropharyngeal structures (i.e., soft palate, base of tongue, and epiglottis) is a definite risk in patients with obesity after anesthesia induction or sedation^18^ during which a patent airway remains a key factor for successful oxygenation^19^, the results of the previous meta-analysis^17^ may not be applicable to those with obesity. To clarify the benefits of HFNO in this patient population, this meta-analysis aimed at comparing the risk of hypoxemia, oxygenation status, and carbon dioxide elimination between patients with obesity receiving HFNO and those undergoing conventional oxygen therapy (COT) or non-invasive ventilation (NIV) in a variety of clinical settings.

## Methods

This meta-analysis was conducted in accordance with the recommendations of the PRISMA statement and registered with the International Prospective Register of Systematic Reviews (CRD42021271777).

### Data sources and searches

We searched the Embase, Medline, Google scholar, and the Cochrane Library databases from inception to August 19, 2021, using the following search terms: ("Obesity" or "Obes*" or "Overweight" or "Severe Obesity" or "Morbid Obesity") and ("(high flow or high-flow) ADJ4 (oxygen or cannula* or oxygenation)" or "HFNO" or "HFNC" or "NHF" or "Optiflow" or "THRIVE" or "Transnasal Humidified Rapid Insufflation Ventilatory Exchange") limited to randomized controlled trials (RCTs). No restriction was placed on language, gender, sample size, and study location during literature search. The search strategies for these databases are demonstrated in Supplemental Table 1. Regarding Google scholar, a hand-search strategy was adopted to find the related articles. Once a relevant article was identified, a forward snowballing strategy^20,21^ was used to optimize the efficiency of the literature search. Additional records identified by reviewing the reference lists of the retrieved studies were also reviewed for eligibility of being included in the current study.

### Inclusion criteria

To scrutinize the eligibility of the acquired publications for the present meta-analysis, we adopted the following PICO (population, intervention, comparison, outcomes) criteria: (a) Population: adults patients (age ≥ 18 years) with obesity, (b) Intervention: the use of HFNO as the intervention measure, (c) Comparison: the use of COT [e.g., mask/nasal oxygenation] or NIV as a control, (d) Outcomes: inclusion of at least one of these outcomes: incidence of hypoxemia, minimum O_2_ saturation, PaO2, safe apnea time, PaCO_2_ or EtCO_2_. Only RCTs were included for analysis. The authors of the included articles with missing information were contacted for possible access to the original data.

### Exclusion criteria

Exclusion criteria were: (1) studies without a control group; (2) those focusing on patients undergoing cardiothoracic surgeries; (3) those in which information regarding outcomes was unavailable, and (4) RCTs published only as letters or abstracts, or (5) those presented as a review, case report, or other forms of publication other than original research.

### Study selection

Two authors examined the titles and abstracts of the retrieved RCTs independently for eligibility of being included in the present study. The full texts of the potentially eligible trials were independently reviewed based on the inclusion and exclusion criteria. Differences in opinions about inclusion or exclusion of a particular study were resolved through discussion with a third reviewer.

### Data extraction

The following items were retrieved from each trial: first author, year of publication, age, gender, body mass index (BMI), sample size, flow rate of HFNO, type of surgery or procedures, incidence of hypoxemia, level of PaO_2_, minimum O_2_ saturation, safe apnea time, EtCO_2_, and PaCO_2_. Disagreements were settled by discussion with a third author.

### Outcomes and definitions

The primary outcome was the impact of HFNO on the risk of hypoxemia, while the secondary outcomes included minimum SaO_2_, level of PaO_2_, PaO_2_/FiO_2_ ratio, EtCO_2_, PaCO_2_, and safe apnea time. The definition of hypoxemia was in accordance with that of each study. As the efficacy for oxygenation may be different between COT and NIV, subgroup analysis of the impact of choosing either approach as control to assess the therapeutic benefit of HFNO was performed. If a study reported an outcome (e.g., level of PaO_2_) at different time points, we analyzed the data acquired just before invasive mechanical ventilation.

### Assessment of risk of bias

Internal validity of the included RCTs was assessed by two reviewers independently based on the following domains: adequacy of sequence generation, allocation sequence concealment, blinding of participants and caregivers, blinding for outcome assessment, incomplete outcome data, selective outcome reporting, and the other sources of bias^22^. The risk of bias of each RCT was reported as "low," "unclear", or "high". We regarded the risk of "selective outcome reporting" bias of a study as "unclear" if its protocol was not published or registered. Moreover, the sources of funding were scrutinized for the potential of other biases. Disagreements were resolved by discussion.

### Data synthesis and analysis

Cochrane Review Manager (RevMan 5.3; Copenhagen: The Nordic Cochrane Centre, The Cochrane Collaboration, 2014) was used for the present meta-analysis. The pooled risk ratios (RRs) and mean difference (MD) with 95% confidence intervals (CIs) were computed for binary and continuous outcomes, respectively. We assessed heterogeneity with I^2^ statistics and defined substantial heterogeneity as an I^2^ over 50%. On the assumption of heterogeneity across the included studies, we adopted a priori a random-effects model for outcome evaluation. The potential publication bias was assessed by visual inspection of a funnel plot on encountering 10 or more trials sharing a particular outcome. Sensitivity analysis was conducted with a leave-one-out approach to weigh the potential influence of the data from an individual trial on the overall outcome. The level of significance was set at < 0.05 for all outcome analyses.

## Results

### Search results and study characteristics

**Figure 1:**
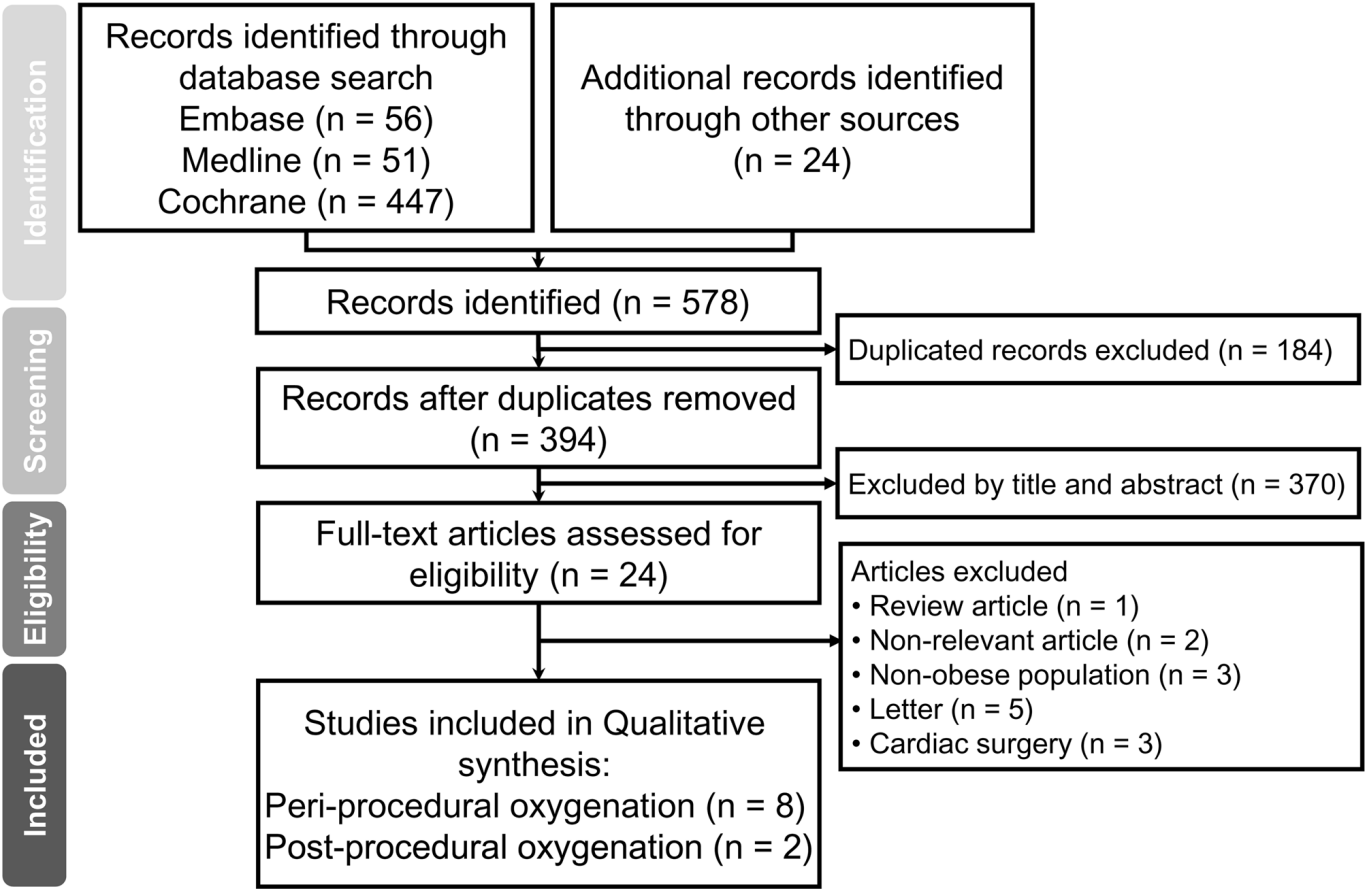
PRISMA flow diagram of study selection for the current meta-analysis.

The process of study selection is shown in Fig. 1. First, of the 578 potentially relevant records retrieved from the databases, 184 duplicates were excluded. Second, screening of the titles and abstracts based on the PICO criteria gave 24 potentially eligible studies. Finally, after all text reviews, 10 studies published from 2014 to 2021 involving 564 patients were included in current meta-analysis. Characteristics of the included studies are demonstrated in Table 1. All studies recruited participants of both genders with the proportion of females ranging from 29.7 to 89.5%. The mean BMI in the enrolled patients varied from 33 to 52 kg/m^2^. The proportion of patients with obstructive sleep apnea (OSA) was between 12.5 and 72.5% in five trials^23–27^, while five studies did not provide this information^28–32^. Of the ten studies, two assessed the beneficial effects of HFNO on postoperative pulmonary parameters in patients receiving laparoscopic bariatric surgery^23,24^, while eight studies evaluated the efficacy of HFNO against peri-procedural hypoxemia or oxygenation status during anesthesia induction (six trials)^26–30,32^, tracheal intubation in the intensive care unit (one trial)^31^, and colonoscopy under deep sedation (one trial)^25^. In the HFNO groups, the flow rate ranged from 50 to 120 L/min. In the control groups receiving COT/NIV, mask oxygenation was adopted in four studies^23,24,27,29^, while NIV and nasal cannula oxygenation were used in other four^26,30–32^ and two^25,28^ RCTs, respectively.

**Table 1:** Characteristics of included studies (n = 10).

Studies	Mean Age (years) H vs. C	Sample size	Female (%)	BMI (kg/m^2^) H vs. C	OSA (%)	Procedure	Setting	Flow (H)	Flow (C)	Country
Ferrando 2019	46.3 vs. 46.4	64	66	43.1 vs. 45.5	12.5	LBS	PO	60 L/min; FiO_2_ = 0.5	MO at 15 L/min; FiO_2_ = 0.5	Spain
Fulton 2021	48 vs. 46	50	78	43.1 vs. 44.4	46	LBS	PO	50 L/min; FiO_2_ = 0.5	MO 6 L/min	Australia
Hamp 2020	47 vs. 40	40	72.5	46.3 vs. 45.8	NA	Bariatric surgery	PPO	120 L/min	NCO at 10 L/min	Austria
Heinrich 2014	41 vs. 47†	22	55	52 vs. 46	NA	LBS	PPO	50 L/min; FiO_2_ = 1	MO at 12 L/min; FiO_2_ = 1	Germany
Jiang 2020	47.1 vs. 46.5	60	48.3	33 vs. 33.9	NA	LC	PPO	70 L/min, FiO_2_ = 1	NIV; FiO_2_ = 1	China
Riccio 2019	54 vs. 59	59	86.4	48 vs. 49	16.9	Colonoscopy¶	PPO	60 L/min; FiO_2_ = 0.36–0.4	NCO at 4 L/min; FiO_2_ = 0.36–0.4	United States
Rodriguez 2021	66 vs. 66	91	29.7	34 vs. 35	NA	TI‡	PPO	60 L/min; FiO_2_ = 1	NIV; PEEP = 5 cmH2O; FiO_2_ = 1	French
Rosen 2021	44 vs. 38.7	38	89.5	39.8 vs. 40	13.2	LBS	PPO	70 L/min, FiO_2_ = 1	NIV; PEEP = 7 cmH2O; FiO_2_ = 1^a^	Sweden
Vourch 2019	51 vs. 46†	100	70	42 vs. 41	NA	Mixed surgery	PPO	60 L/min, FiO_2_ = 1	NIV; PEEP = 5 cmH2O; FiO_2_ = 1	France
Wong 2019	43.1 vs. 44	40	77.5	48.7 vs. 48.8	72.5	NA	PPO	60 L/min, FiO_2_ = 1	MO at 15 L/min; FiO_2_ = 1	Canada

*H* high-flow nasao oxygenation group, *C* control group, vs. C †data were presented as median, *LBS* laparoscopic bariatric surgery, ¶ procedure was performed under deep sedation, *MO* mask oxygenation, *FiO*_*2*_ fraction of inspired oxygen, *NCO* nasal cannula oxygenation, *MV* mask ventilation, *BMI* body mass index, *TI* tracheal intubation, ‡ performed in intensive care units, *PO* post-procedure oxygenation supplementation, *PPO* peri-procedure oxygenation supplementation, *NIV* noninvasive ventilation.

### Risk of bias assessment

**Figure 2:**
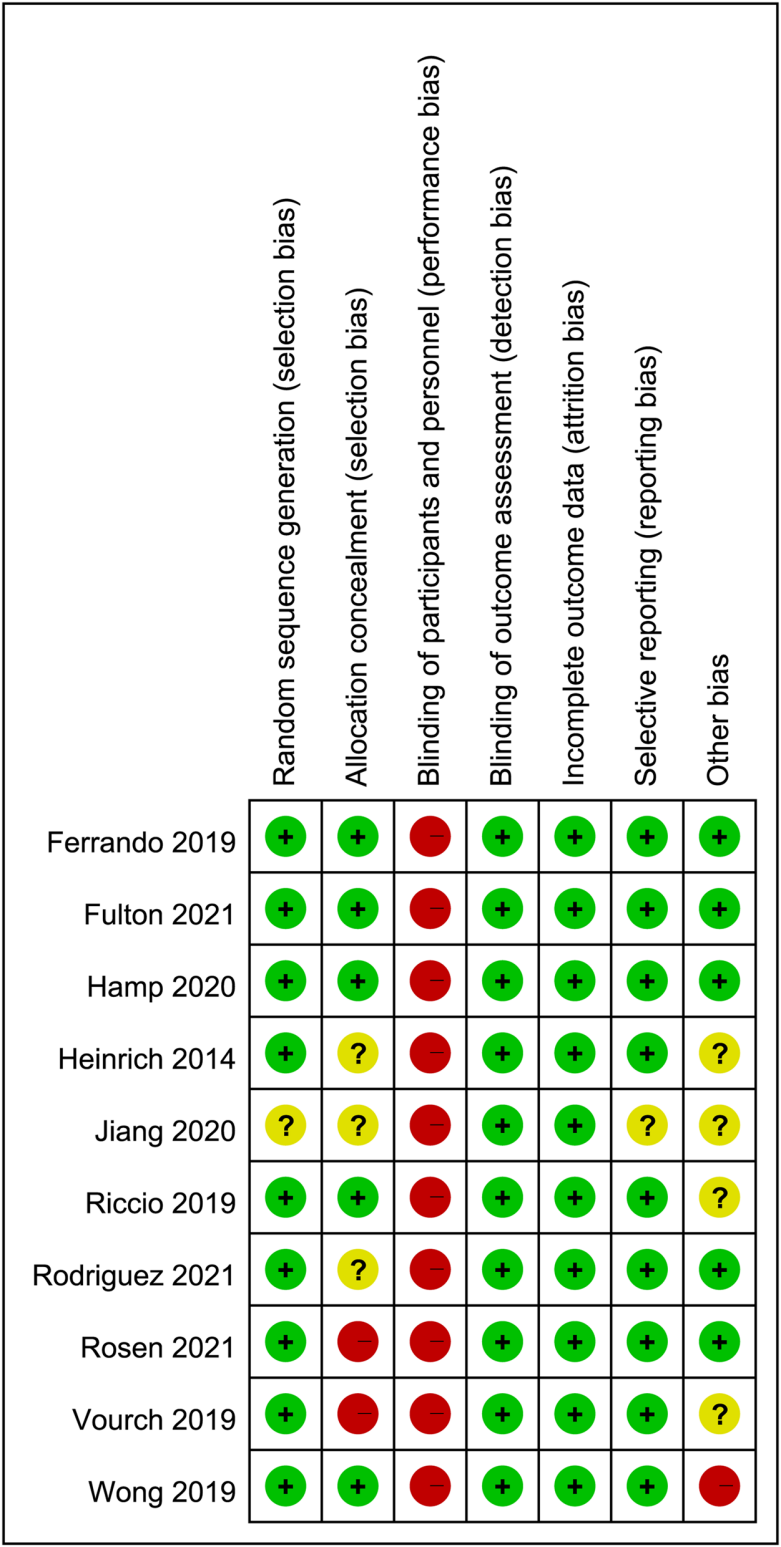
Risks of bias of the included studies.

The risks of bias of individual studies are presented in Fig. 2. Regarding selecting bias, the risks of bias were low in most studies. However, one study did not give information about randomization of patients^30^, three RCTs did not described the methods used to accomplish allocation concealment^29–31^, and two trials reported that the allocation concealment was not masked^26,32^. Considering the impossibility of blinding among patients and caregivers in the included trials, performance bias was high in all studies^23–32^. Despite the lack of blinding, the risk of detection bias was considered low in all studies that used objective indicators (e.g., PaO_2_) as assessment parameters. The risk of reporting bias was unclear in one study^30^ that did not declare trial registration, while the risk of other biases was unclear or high in five studies^25,27,29,30,32^.

### Outcomes analyses

#### Primary outcome: impact of HFNO on risk of hypoxemia

**Figure 3:**
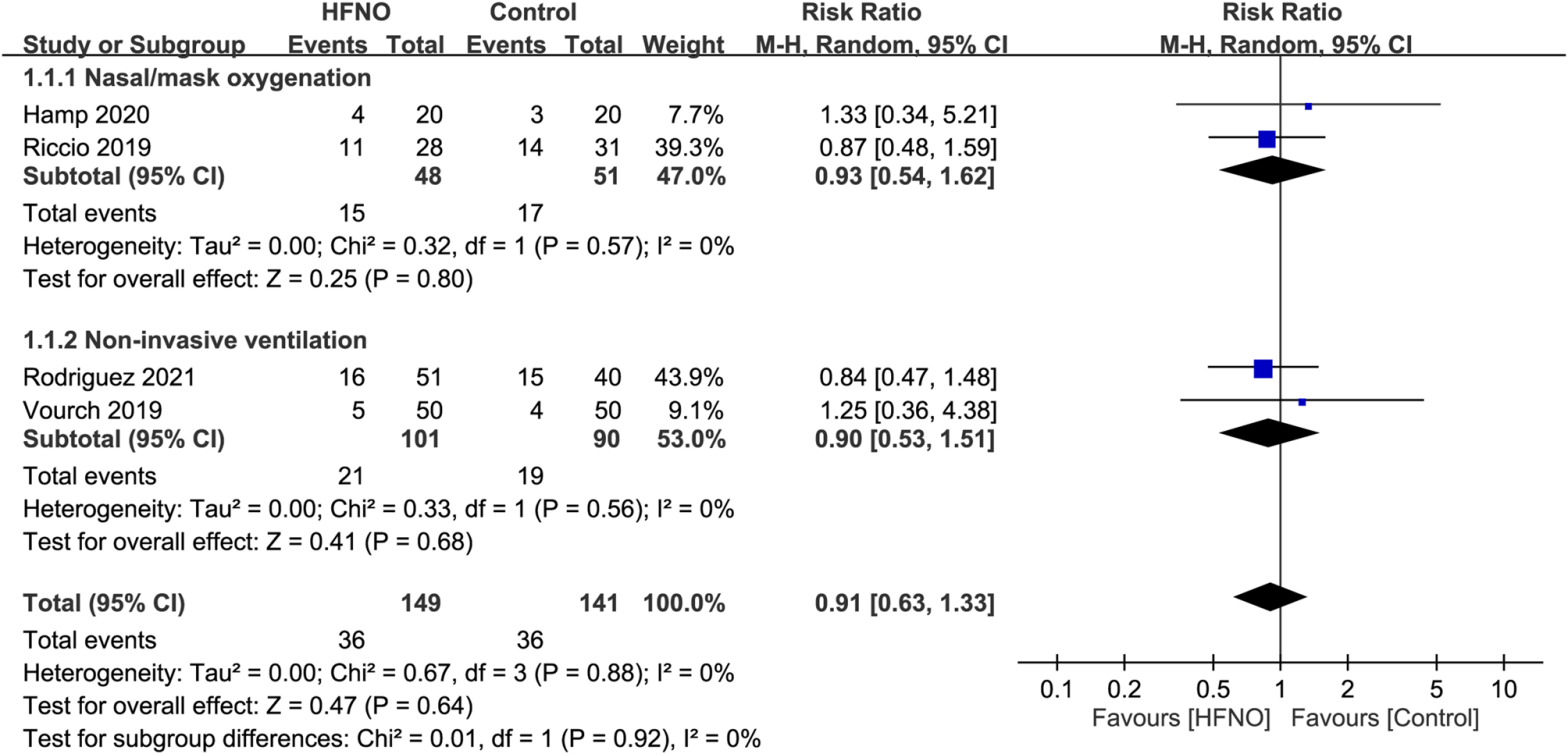
Forest plot comparing the risk of hypoxemia between HFNO and control groups. HFNO, high-flow nasal oxygenation; M-H, Mantel–Haenszel; CI, confidence interval.

Of the four RCTs included in the present meta-analysis, hypoxemia was defined as an SaO_2_ < 90% in three studies^25,28,32^, and < 80% in one trial^31^ which was conducted in the intensive care unit. The incidence of hypoxemia was 24.2% and 25.5% in the HFNO and control group, respectively. Pooled results revealed no significant difference in the risk of hypoxemia between patients receiving HFNO and those undergoing COT/NIV (RR = 0.91, 95% CI 0.63 to 1.33, *p* = 0.64; I^2^ = 0%; 4 RCTs; n = 290) (Fig. 3). Consistently, subgroup analysis showed no significant impact of the choice of different approaches (i.e., COT or NIV) on the risk of hypoxemia (*p* = 0.92) (Fig. 3). Sensitivity analysis demonstrated a consistent finding when the four trials were removed one at a time.

#### Secondary outcome: impact of HFNO on peri-procedural oxygenation-related parameters

**Figure 4:**
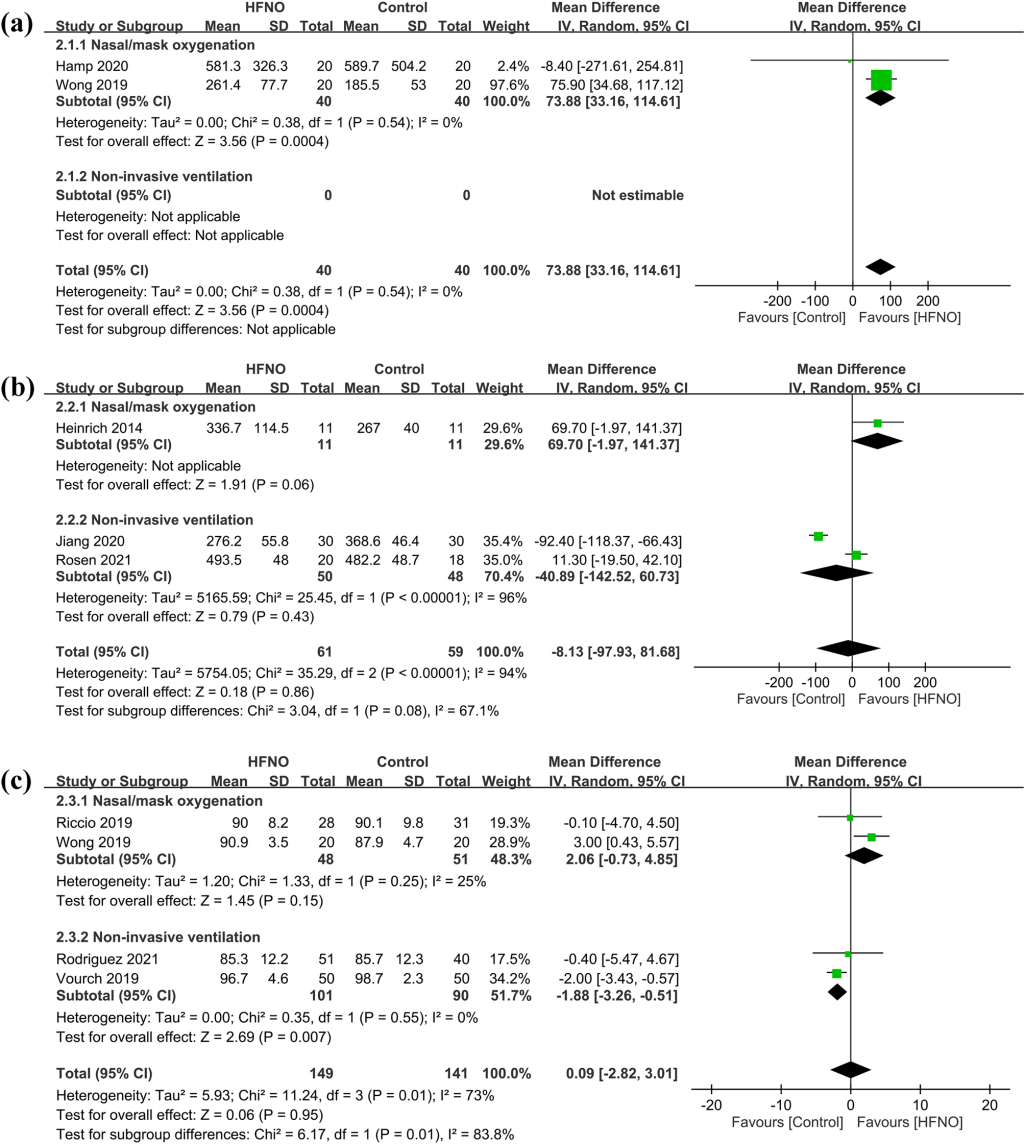
Forest plot comparing (**a**) safe apnea time, (**b**) PaO_2_, and (**c**) minimum SpO_2_ between HFNO and control groups. HFNO, high-flow nasal oxygenation; IV, inverse variance; CI, confidence interval.

The safe apnea time was defined in two of the included studies either as the time taken for the SpO_2_ to drop below 95%^27,28^ or the maximum time of observation before invasive mechanical ventilation, which was six minutes in one study^27^ and 15 min in the other^28^. Our results demonstrated that the use of HFNO significantly increased the safe apnea time compared to the use of COT (MD = 73.88 s, 95% CI 33.16–114.61, *p* = 0.0004; I^2^ = 0%; 2 RCTs; n = 80) (Fig. 4a)^27,28^. However, forest plot showed no significant difference in PaO_2_ (MD = − 8.13 mmHg, 95% CI − 97.93 to 81.68, *p* = 0.86; I^2^ = 94%; 3 RCTs; n = 120) between the two groups (Fig. 4b)^26,29,30^. Sensitivity analysis demonstrated a consistent finding when one trial was removed one at time. In addition, forest plot also revealed no significant difference in minimum SpO_2_ (MD = 0.09%, 95% CI − 2.82 to 3.01, *p* = 0.95; I^2^ = 73%; 4 RCTs; n = 290) between the two groups (Fig. 4c)^25,27,31,32^. Sensitivity analysis indicated that the minimum SpO_2_ was lower in the HFNO group compared to that in the control group when one study^27^ was removed.

Subgroup analysis showed no significant impact of the choice of different approaches (i.e., COT or NIV) on PaO2 (*p* = 0.08) (Fig. 4b). For minimum SpO_2_, subgroup analysis revealed a significantly lower minimum SpO_2_ associated with the use of HFNO compared to that with NIV (MD = − 1.88%, 95% CI − 3.26 to − 0.51, *p* = 0.007; I^2^ = 0%; 2 RCTs; n = 191), while there was no difference between HFNO and COT (*p* = 0.15).

#### Secondary outcome: impact of HFNO on peri-procedural carbon dioxide level

**Figure 5:**
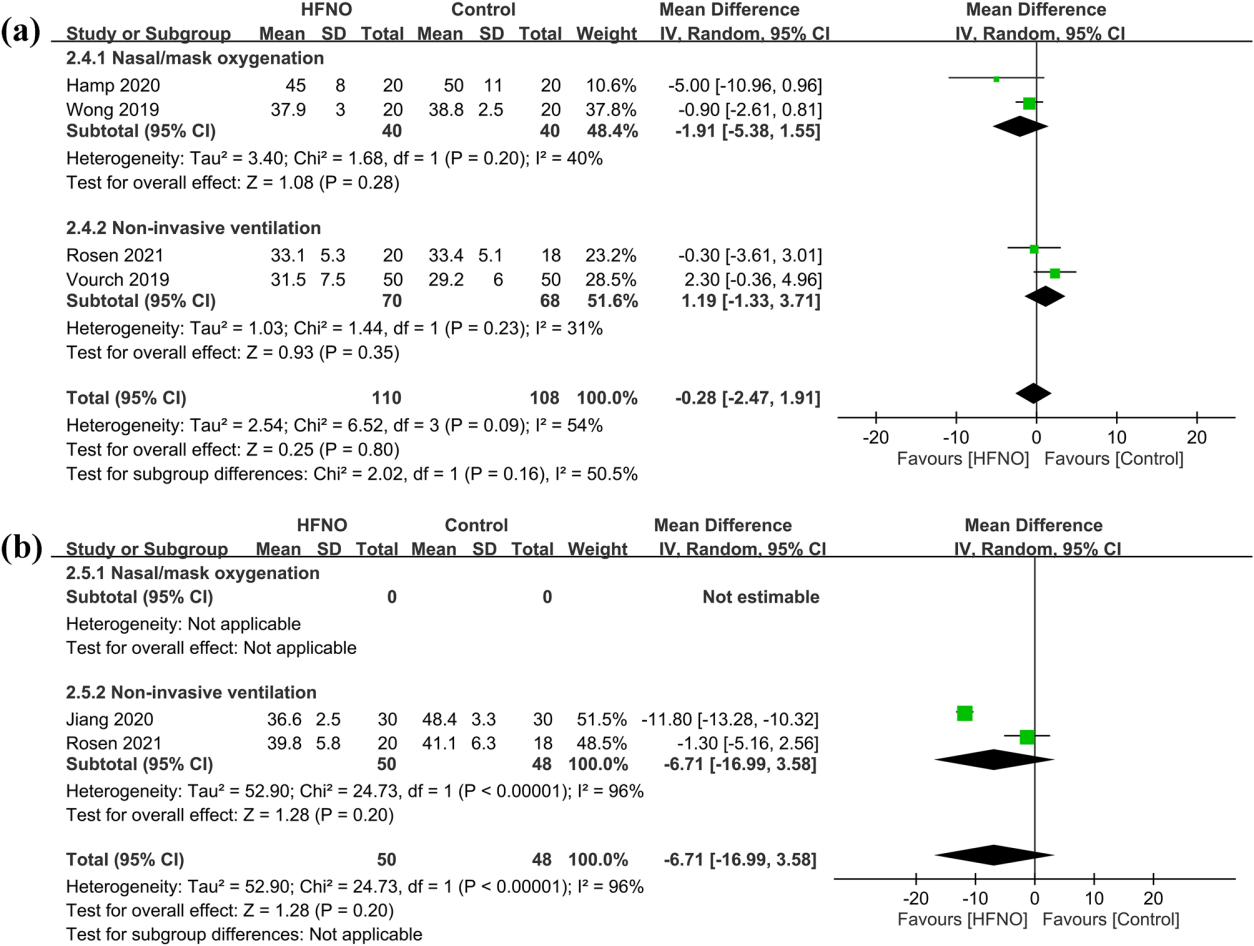
Forest plot comparing (**a**) EtCO_2_ and (**b**) PaCO_2_ between HFNO and control groups. HFNO, high-flow nasal oxygenation; IV, inverse variance; CI, confidence interval.

Our results demonstrated no significant difference in EtCO_2_ (MD = − 0.28 mmHg, 95% CI − 2.47 to 1.91, *p* = 0.8; I^2^ = 54%; 4 RCTs; n = 218) (Fig. 5a)^26–28,32^ between the HFNO and control groups. Sensitivity analysis verified a consistent finding when one trial was removed one at time. Forest plot showed no significant difference in the PaCO_2_ (MD = − 6.71%, 95% CI − 16.99 to 3.58, *p* = 0.2; I^2^ = 96%; 2 RCTs; n = 98) (Fig. 5b)^26,30^ between the HFNO and control groups. The limited availability of trials (i.e., only two) precluded the conduction of a sensitivity analysis of this outcome. Subgroup analysis indicated no significant impact of choosing different approaches of conventional oxygenation (i.e., COT or NIV) on EtCO2 (*p* = 0.16) (Fig. 5a).

#### Secondary outcome: impact of HFNO on postprocedural respiratory parameters

**Figure 6:**
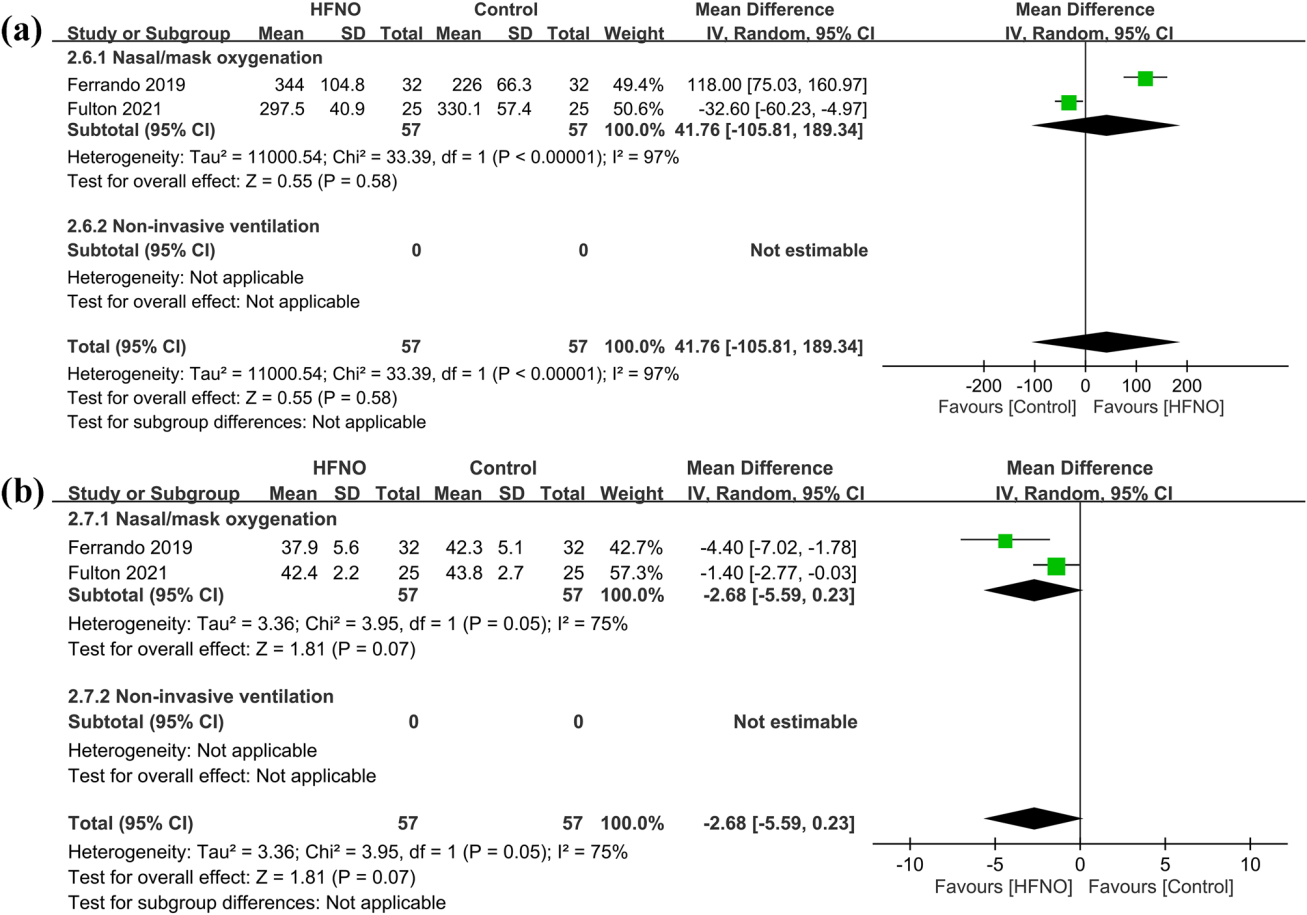
Forest plot comparing (**a**) PaO_2_/FiO_2_ ratio and (**b**) PaCO_2_ level between HFNO and control groups. HFNO, high-flow nasal oxygenation; IV, inverse variance; CI, confidence interval.

Two RCTs provided information on postprocedural respiratory parameters at three hours^23,24^. This time point was chosen based on the observation that patients undergoing bariatric surgery usually spend three hours in the postanesthesia care unit before discharge^23^. Forest plot revealed comparable respiratory parameters, namely, PaO_2_/FiO_2_ ratio (MD = 41.76, 95% CI − 105.81 to 189.34, *p* = 0.58; I^2^ = 97%; 2 RCTs; n = 114) (Fig. 6a) and PaCO_2_ (MD = − 2.68 mmHg, 95% CI − 5.59 to 0.23, *p* = 0.07; I^2^ = 75%; 2 RCTs; n = 114) between the HFNO and control groups (Fig. 6b). Sensitivity analysis was not performed because only two trials were available for outcome comparison.

## Discussion

Despite oxygen supplementation, patients with obesity may still experience significant hypoxemia after anesthesia-induced apnea because of a reduced FRC and an increased minute oxygen demand^11^ that highlight the importance of implementing appropriate postprocedural oxygenation strategy for improving patient safety. Accordingly, the current meta-analysis focused on a comparison between HFNO and COT/NIV in this particular patient population**.** Our meta-analysis demonstrated that the use of HFNO prolonged the apnea time without a beneficial impact on the risk of hypoxemia, minimum SpO_2_, PaO_2_, EtCO_2_, and PaCO_2_ in patients with obesity receiving peri-procedural oxygenation. After tracheal extubation, the application of HFNO was also not associated with an elevated PaO_2_/FiO_2_ ratio and a decreased PaCO_2_ level at postoperative three hours.

Although a previous meta-analysis showed that the use of HFNO could reduce the risk of hypoxemia in patients receiving sedation or anesthetic induction^17^, only one of the included trials recruited patients with obesity; therefore, its findings may not be applicable to patients with obesity. Indeed, our results did not support a superior beneficial effect of HFNO against hypoxemia compared to that in the control group. Consistently, the levels of PaO_2_ and minimum SpO_2_ were comparable between the two groups, indicating no significant association between the use of HFNO and a reduced risk of hypoxemia. Therefore, one of the striking clinical implications of the present study was that this patient population, who are at risk of hypoxemia, may not benefit from the use of HFNO. In concert with our finding, a closed claims analysis on the management of difficult tracheal intubation showed that a delay in alternative airway intervention and judgment errors may contribute to brain ischemia and mortality^33^. Besides, the use of HFNO may be associated with an elevated risk of delayed airway management (e.g., tracheal intubation)^34^ possibly because of a false sense of security that loosens the alert for potential airway problems. In this regard, we suggest that hypoxemia in patients with obesity receiving oxygen supplementation with HFNO should be promptly managed without exposing the patients to unnecessary risks.

A recent international multicenter trial comparing HFNO with standard facemask pre-oxygenation for rapid sequence induction in patients with a normal body build (i.e., mean BMI around 25 kg/m^2^)^35^ demonstrated no difference in the incidence of hypoxemia (i.e., SpO_2_ < 93%) between pre-oxygenation using HFNO or tight facemask. Our findings were consistent with those in that study^35^. The lack of efficacy of HFNO for the prevention of hypoxemia compared to COT/NIV may be attributable to inadequate positive airway pressure associated with HFNO. First, although a previous study suggested the need for an adequate airway patency to achieve effective oxygenation^32^, the limited positive airway pressure generated by HFNO (e.g., 2.7 cmH_2_O) may be unable to relieve airway obstruction after anesthesia or sedation in patients with obesity^36^. Moreover, although a previous study has demonstrated a positive correlation between the flow rate of HFNO and nasopharyngeal pressure, which could reach over 3 cmH_2_O at a flow rate of 50 L/min^37^, whether a higher flow could improve the risk of hypoxemia in patients with obesity remains unclear. In the current study, there were four trials that provided the outcome of hypoxemia. While three of the trials^25,31,32^ used a flow rate of 60 L/min, the other^28^ adopted a flow rate of 120 L/min. Despite the obvious difference, our sensitivity analysis demonstrated that removal of the study using a higher flow rate^28^ had no significant impact on the risk of hypoxemia. Nevertheless, since the number of trials included in the current meta-analysis was relatively small to arrive at a robust conclusion. Second, maintenance of an adequate FRC and avoidance of alveolar collapse is also important for efficient oxygenation^32^. Although a previous small-scale study with 20 participants reported an increased lung volume and FRC as another potential benefit of HFNO particularly in patients with higher BMIs^38^, that study included only two patients with BMI > 40 kg/m^2^. In contrast, all of our included studies focused on patients with BMI > 30 kg/m^2^. Therefore, our findings implicated that the low-level positive airway pressure generated by HFNO may not be able to increase the lung volume in our patient population.

Apart from the lack of a beneficial influence of HFNO on hypoxemia, the present study also showed no positive impact of HFNO on peri-procedural CO_2_ clearance. Although a previous study demonstrated that the enhanced CO_2_ clearance associated with the use of HFNO may be flow-dependent^39^, our results (flow: 50–120 L/min) and those of a recent study (flow: 70 L/min)^35^ did not support this finding that the use of HFNO was associated with a low CO_2_ clearance. Regarding the impact of HFNO on postprocedural CO_2_ clearance, our results were derived from two trials that recruited patients undergoing laparoscopic operations in which CO2 was used for abdominal CO_2_ insufflation. Although CO_2_ clearance may be modified by anesthesiologists immediately after laparoscopic surgery, the present study focused on postoperative three hours so that such an impact would be minimal. Nevertheless, our findings may not be extrapolated to patients receiving non-laparoscopic procedures. Further studies are needed to address this issue.

One recent meta-analysis of three clinical trials enrolling 160 patients with or without obesity reported a safe extension of apnea time by 33.4 s in participants receiving HFNO versus those subjected to COT at anesthesia induction^17^. In spite of the demonstration of a HFNO-associated prolongation of safe apnea time compared to COT in the current study, the finding should be interpreted with caution. First, in spite of our finding of a significant prolongation of safe apnea time, the result was based on two trials enrolling only 80 patients^27,28^. Second, a prolongation of merely 73 s may not be of clinical significance in patients with obesity who usually present with a difficult airway^9,10^. Third, notwithstanding the inclusion of patients with similar BMI and age, there were wide variations between the two studies^27,28^ in mean apnea time in both the HFNO (i.e., 581.3^28^ vs. 261.4^27^ s) and COT (i.e., 589.7^28^ vs. 185.5^27^ s) groups. Because of the heterogeneity, more studies are required to explore the efficacy of HFNO for prolonging safe apnea time. Overall, our results are in line with those of a recent study^35^ that reported comparable incidences of hypoxemia between patients with or without HFNO despite a prolonged safe apnea in those receiving HFNO^35^.

In patients undergoing bariatric surgery, the prevalence of postoperative atelectasis could be as high as 37%^40^. Not only does obesity predispose to postoperative atelectasis but atelectasis in this patient population also resolves more slowly than in those with normal body build^41,42^. Despite the recommendation of oxygen supplementation in patients at high risk of postoperative atelectasis^43^, we found that the use of HFNO was unable to improve oxygenation parameters in patients with obesity both during the periprocedural period and at three hours after surgery. Consistent with our findings, another meta-analysis investigating patients with obesity undergoing cardiac surgery demonstrated no significant improvements in atelectasis score, dyspnea score, PaO_2_/FiO_2_ ratio, and reintubation rate in patients receiving HFNO compared with those undergoing COT^44^. The lack of benefits of HFNO in patients with obesity during the postoperative period underscores the need for timely intervention (e.g., reintubation) in case of respiratory distress after tracheal extubation.

Our study had several limitations. First, the limited number of trials not only blemished the reliability of our findings but also precluded our analysis of some clinical outcomes such as the risk of atelectasis or hypoxemia-associated complications. Second, the heterogeneity across the included trials in study settings (e.g., anesthetic induction vs. sedation, intensive care unit vs. operating theater) and approaches (i.e., COT or NIV) may introduce bias to our results. Nevertheless, a comparison between HFNO and other widely accepted clinical approaches to oxygen supplementation (e.g., COT and NIV) is still of clinical significance. Third, our findings on the association between HFNO and conventional respiratory parameters may not reflect clinical outcomes. Fourth, all included studies in the current meta-analysis investigated patients with BMI > 30 kg/m^2^, the efficacy of HFNO against hypoxemia in patients with less severe obesity remains to be elucidated.

## Conclusion

The current study showed that, compared with conventional oxygen therapy or non-invasive ventilation, the use of high-flow nasal oxygenation was unable to provide additional peri- or post-procedural respiratory benefits for patients with obesity (i.e., BMI > 30 kg/m^2^) except for a prolongation of safe apnea time. Further large-scale studies are warranted to validate our findings.

## Supplementary Information

Supplementary Information.
