# Supplementary material for: Efficacy of high-flow nasal oxygenation against peri- and post-procedural hypoxemia in patients with obesity: a meta-analysis of randomized controlled trials
Source: Sci Rep. 2022 Apr 19;12:6448. doi: 10.1038/s41598-022-10396-5 (PMC9018711; doi:10.1038/s41598-022-10396-5)
Supplement: Supplementary file 1 — Supplementary Information. [file 41598_2022_10396_MOESM1_ESM.docx]

**Supplemental table 1. Search strategies for databases**

|  | **Medline** |
| --- | --- |
| 1 | ("Obesity" or "Obes*" or "Overweight" or "Severe Obesity" or "Morbid Obesity").mp. |
| 2 | exp "obesity"/ or exp "Obesity, Morbid"/ or exp "Overweight"/ |
| 3 | ("(high flow or high-flow) ADJ4 (oxygen or cannula* or oxygenation)" or "HFNO" or "HFNC" or "NHF" or "Optiflow" or "THRIVE" or "Transnasal Humidified Rapid Insufflation Ventilatory Exchange").mp. |
| 4 | (1 or 2) and 3 |
| 5 | 4 and (((randomized controlled trial or controlled clinical trial).pt. or randomi*ed.ab. or placebo.ab. or drug therapy.fs. or randomly.ab. or trial.ab. or groups.ab.) not (exp animals/ not humans.sh.)) |
|  | **Embase** |
| 1 | ("Obesity" or "Obes*" or "Overweight" or "Severe Obesity" or "Morbid Obesity"):ti,ab,kw,de |
| 2 | "obesity"/exp or "Obesity, Morbid"/exp or "Overweight"/exp |
| 3 | ("(high flow or high-flow) ADJ4 (oxygen or cannula* or oxygenation)" or "HFNO" or "HFNC" or "NHF" or "Optiflow" or "THRIVE" or "Transnasal Humidified Rapid Insufflation Ventilatory Exchange"):ti,ab,kw,de |
| 4 | (#1 OR #2) AND #3 |
| 5 | #4 AND ('crossover procedure':de OR 'double-blind procedure':de OR 'randomized controlled trial':de OR 'single-blind procedure':de OR (random* OR factorial* OR crossover* OR cross NEXT/1 over* OR placebo* OR doubl* NEAR/1 blind* OR singl* NEAR/1 blind* OR assign* OR allocat* OR volunteer*):de,ab,ti) |
|  | **Cochrane library** |
| 1 | ("Obesity" or "Obes*" or "Overweight" or "Severe Obesity" or "Morbid Obesity"):ti,ab,kw |
| 2 | [mh "obesity"] or [mh "Obesity, Morbid"] or [mh "Overweight"] |
| 3 | ("(high flow or high-flow) ADJ4 (oxygen or cannula* or oxygenation)" or "HFNO" or "HFNC" or "NHF" or "Optiflow" or "THRIVE" or "Transnasal Humidified Rapid Insufflation Ventilatory Exchange"):ti,ab,kw |
| 4 | (#1 OR #2) AND (#3) |

**Supplemental table 2. Risk of bias judged by authors**

| **Study** | **Item** | **Risk** | **Judgement** |
| --- | --- | --- | --- |
| **Ferrando 2019** | Random sequence generation | Low | Participating patients were randomly and blindly assigned to either HFNC or standard-care oxygen therapy using a computer-generated table of codes and individual closed-sealed envelopes. |
|  | Allocation concealment | Low | Participating patients were randomly and blindly assigned to either HFNC or standard-care oxygen therapy using a computer-generated table of codes and individual closed-sealed envelopes. |
|  | Blinding of participants and personnel | High | No specific statement. |
|  | Blinding of outcome assessment | Low | Postoperative data, i.e., from the ward, were collected by an investigator who was blinded to the patients’ randomization arm (HFNC or Standard). |
|  | Incomplete outcome data | Low | No patients were excluded. |
|  | Selective reporting | Low | ClinicalTrials.gov NCT03155711. |
|  | Other bias | Low | The authors certify that there is no conflict of interest. |
| **Fulton 2021** | Random sequence generation | Low | Patients were randomised in a 1:1 fashion (using a computer-generated sequence based on block sizes of 4, 6, or 8) |
|  | Allocation concealment | Low | An investigator, not involved in the perioperative care of a patient, obtained the allocation in a sealed envelope and informed the intensive care unit staff. |
|  | Blinding of participants and personnel | High | The surgical and anaesthetic teams were blinded to the patient’s allocation until return to the ICU, after which treatment allocation was unblinded. |
|  | Blinding of outcome assessment | Low | The surgical and anaesthetic teams were blinded to the patient’s allocation until return to the ICU, after which treatment allocation was unblinded. |
|  | Incomplete outcome data | Low | All patient were followed. |
|  | Selective reporting | Low | ACTRN12617000694314. |
|  | Other bias | Low | Authors have no conflict of interests. |
| **Hamp 2020** | Random sequence generation | Low | Randomization was performed with block randomization using blocks of 10 patients based on a computer-derived randomization list. |
|  | Allocation concealment | Low | Information about the group allocation was put into opaque, consecutively numbered envelopes. |
|  | Blinding of participants and personnel | High | No specific statement. |
|  | Blinding of outcome assessment | Low | No specific statement. |
|  | Incomplete outcome data | Low | All patient were followed. |
|  | Selective reporting | Low | ClinicalTrials.gov (NCT03550924). |
|  | Other bias | Low | The authors have no conflict of interest. |
| **Heinrich 2014** | Random sequence generation | Low | Randomization and assignment to the groups were carried out by the study coordinator using drawing lots. |
|  | Allocation concealment | Unclear | No specific statement. |
|  | Blinding of participants and personnel | High | No specific statement. |
|  | Blinding of outcome assessment | Low | No specific statement. |
|  | Incomplete outcome data | Low | All patient were followed. |
|  | Selective reporting | Low | German Clinical Trial Register No.: drks00005475. |
|  | Other bias | Unclear | No specific statement. |
| **Riccio 2019** | Random sequence generation | Low | Patients were randomized to HFNC versus standard nasal cannula (SNC) intervention, using a randomization scheme created with SAS version 9.3. |
|  | Allocation concealment | Low | The lists were kept in a locked cabinet in the anesthesia workroom. At the time of enrollment, the study team was unaware of the next intervention and checked the lists only after obtaining informed consent. |
|  | Blinding of participants and personnel | High | No specific statement. |
|  | Blinding of outcome assessment | Low | No specific statement. |
|  | Incomplete outcome data | Low | No patient was excluded from analysis. |
|  | Selective reporting | Low | Clinical Trials Registry :NCT03148262. |
|  | Other bias | Unclear | In kind support was received from Teleflex Incorporated in the form of use of the High-Flow Nasal Cannula unit throughout the duration of the study. |
| **Jiang 2020** | Random sequence generation | Unclear | No specific statement. |
|  | Allocation concealment | Unclear | No specific statement. |
|  | Blinding of participants and personnel | High | No specific statement. |
|  | Blinding of outcome assessment | Low | No specific statement. |
|  | Incomplete outcome data | Low | All patient were followed. |
|  | Selective reporting | Unclear | No specific statement. |
|  | Other bias | Unclear | No specific statement for conflict of interest. |
| **Rodriguez 2021** | Random sequence generation | Low | Randomisation was computer-generated in permuted blocks of four participants (unknown to investigators), with stratification according to the centre and PaO2/FiO2 ratio (≤200 mm Hg and >200 mm Hg). |
|  | Allocation concealment | Unclear | No specific statement. |
|  | Blinding of participants and personnel | High | Patients and operators were not blinded. |
|  | Blinding of outcome assessment | Low | The coordinating centre and all the investigators remained unaware of the outcomes of each study group until the data were locked on Oct 11, 2017. |
|  | Incomplete outcome data | Low | 2.9% of patients were excluded. |
|  | Selective reporting | Low | Trial registration Clinical trial number: NCT02668458 |
|  | Other bias | Low | None delcared. |
| **Rosen 2021** | Random sequence generation | Low | Randomisation was performed with an allocation ratio of 1:1 and a block size of two using sealed opaque sequentially numbered envelopes. |
|  | Allocation concealment | High | There was no masking. |
|  | Blinding of participants and personnel | High | No specific statement. |
|  | Blinding of outcome assessment | Low | No specific statement. |
|  | Incomplete outcome data | Low | All patient were followed. |
|  | Selective reporting | Low | Trial registration: #ISRCTN37375068 (www.isrctn.com). |
|  | Other bias | Low | No conflicts of interest. |
| **Vourch 2019** | Random sequence generation | Low | Patients were randomised in an allocation ratio of 1:1 using varying different blocks sizes. |
|  | Allocation concealment | High | There was no masking strategy. |
|  | Blinding of participants and personnel | High | No specific statement. |
|  | Blinding of outcome assessment | Low | No specific statement. |
|  | Incomplete outcome data | Low | All patient were followed. |
|  | Selective reporting | Low | Clinicaltrials.gov identifier: NCT03106441. |
|  | Other bias | Unclear | Fanny Feuillet, Claire Blanchard, EricMiraille and Christophe Guitton declare no conflict of interest. Mickael Vourc'h declares personal fees from MSD, Pfizer, Baxter and grants from Fischer Paykel, outside the submitted work. Samir Jaber reports personal fees from Draeger, Fresenius-Xenios and Fisher Paykel Healthcare, outside the submitted work. Karim Asehnoune declares personal fees from Fisher Paykel Healthcare, Baxter, LFB, Fresenius. |
| **Wong 2019** | Random sequence generation | Low | Randomization involved computer-generated randomization numbers by a biostatistician and was kept in sealed envelopes. |
|  | Allocation concealment | Low | The envelopes were opened half an hour before the start of the study case by a research coordinator. |
|  | Blinding of participants and personnel | High | No specific statement. |
|  | Blinding of outcome assessment | Low | No specific statement. |
|  | Incomplete outcome data | Low | 11.1% of patients was excluded. |
|  | Selective reporting | Low | Clinicaltrial.gov ID: NCT03195504. |
|  | Other bias | High | There was a research grant from Fisher & Paykel Healthcare Limited. |
